# Supplementary material for: Gas permeation through graphdiyne-based nanoporous membranes
Source: Nat Commun. 2022 Jul 12;13:4031. doi: 10.1038/s41467-022-31779-2 (PMC9276745; doi:10.1038/s41467-022-31779-2)
Supplement: Supplementary file 1 — Supplementary information [file 41467_2022_31779_MOESM1_ESM.pdf]

Gas permeation through graphdiyne-based nanoporous membranes

Supplementary Information

Membrane synthesis and characterization

During synthesis process, Cu foils serve as both catalyst reservoir and planar template for the conformal growth of graphdiyne. Pyridine solvent dissolves Cu ions as catalyst from the surface of Cu foils into solution, and graphdiyne membrane start to occur through the coupling reaction of HEB at copper surface. After growing the first few layers of graphdiyne, consecutive layers continue to grow and stack on the surface of preceding layers by strong π-π interaction, to form a multilayer nano-crystallite structure[1,2] as the bottom layer shown in Fig. 1. As the reaction proceeds, monomers were gradually consumed and monomer's concentration decreases. At this stage, only small amount of isolated graphdiyne nanosheets formed and randomly stacked with each other, even vertically, into some three-dimensional structures on top of the previously formed graphdiyne film. Therefore, two morphologies have been observed from the SEM cross-sectional view as shown in Fig.1.

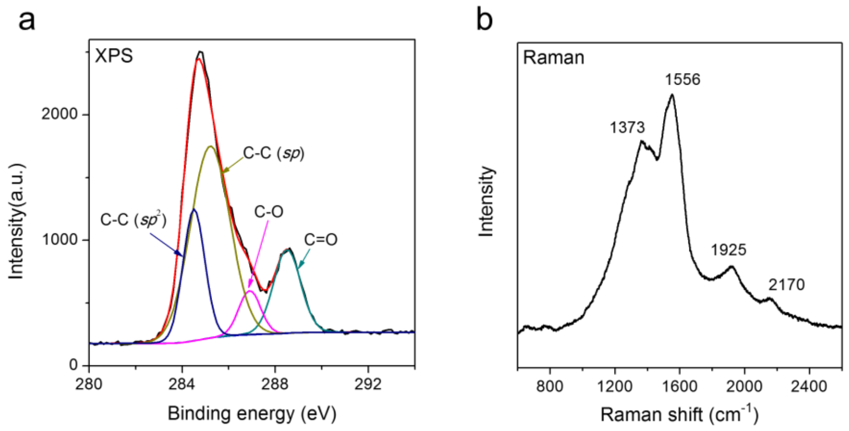

Supplementary Figure 1 | Graphdiyne membrane characterization, using **(a)** XPS and **(b)** Raman spectroscopy techniques. The spectrum peak position assignment in (a) and (b) are in accordance with ref. 1.

The as-synthesized graphdiyne-based membranes were characterized by X-ray photoelectron spectroscopy (XPS) and Raman spectroscopy (Supplementary Figure 1). The results are in consistent with those reported and indicate the presence of graphdiyne structures[1]. In Supplementary Figure 1a, the area ratio of peaks sp/sp^2 is close to 2, in agreement with the graphdiyne structure of benzene rings connected by double -C≡C- links. The presence of C-O and C=O are attributed to air adsorptions. In Supplementary Figure 1b, the peak at 1373 cm^{-1} and 1556 cm^{-1} are attributed to the breathing vibration and stretching modes of sp^2 carbons in aromatic rings, respectively; the peaks at 1925 cm^{-1} and 2170 cm^{-1} correspond to the vibration of conjugated -C≡C-C≡C- bonds.

Morphology characterization

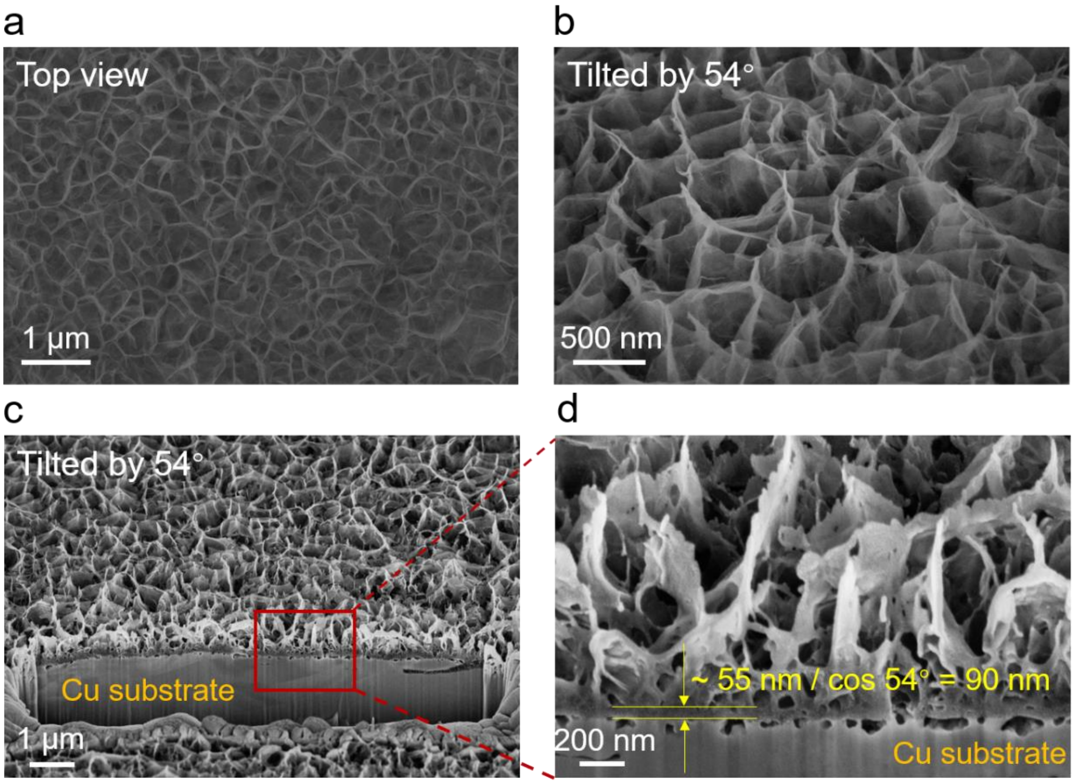

Supplementary Figure 2 | Morphology characterization using electron microscopy. (a) and **(b)** SEM images of as-prepared graphdiyne membranes. **(c)** and **(d)** SEM images at cross-sections prepared by focused ion beam.

Gas transport device fabrication and measurements

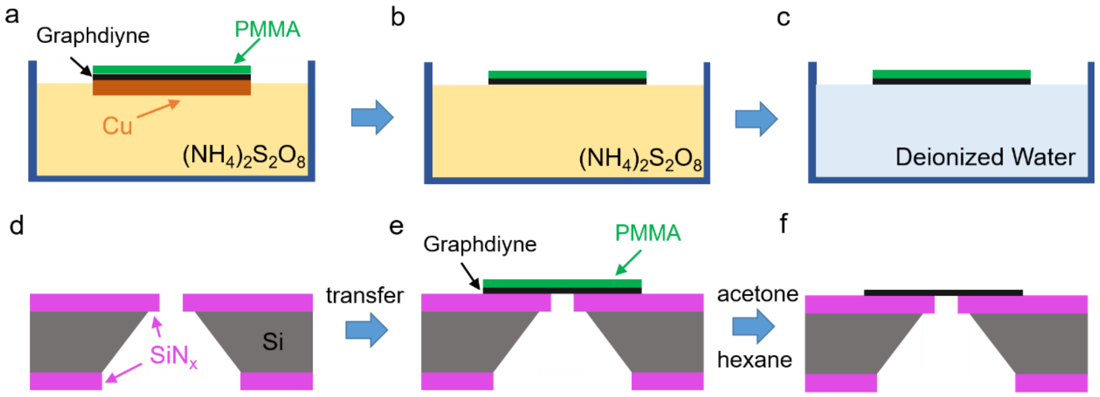

Supplementary Figure 3 | Device fabrication flow. (a) to **(c)** Schematic of copper substrate etching to obtain isolated graphdiyne membrane, similar to that reported in reference 3. **(e)** to **(f)** Schematic of fabrication flow for preparing suspended graphdiyne-based membranes. Detailed aperture fabrication methods and wet transfer methods can be found in reference 4 and 5, respectively.

To calibrate gas permeability measured by the helium leak detector, we note that it allows internal

calibration for the flow rate of ^{4}He. As a control experiment to demonstrate the accuracy of calibration, we tested the permeation of ^{4}He through a 1-μm (in diameter) aperture in silicon nitride. The leak detector correctly measured permeability Γ^*_{4He} that approaches (error < 10%) its theoretical value of $\frac{8}{3N_A}\frac{d_0}{2L}\frac{1}{\sqrt{2\pi mkT}}$, where L is the length of the aperture. In these measurements, the pressure applied is kept < 0.1 bar to ensure the Knudsen condition (mean free path λ > aperture dimension d_0, as discussed in the main texts) is fulfilled. For the calibration of other gas species, a correction factor was calculated from the ratio between their measured permeability and that of ^{4}He (more specifically, $\Gamma^*_{4He} \times \sqrt{\frac{m_{4He}}{m_0}}$, with m_{4He} and m_0 the atomic mass of ^{4}He and gases under investigation, respectively) through the aperture. Similar procedures were applied to the calibration of gas permeation measured by the mass spectrometer, using the same 1-μm aperture.

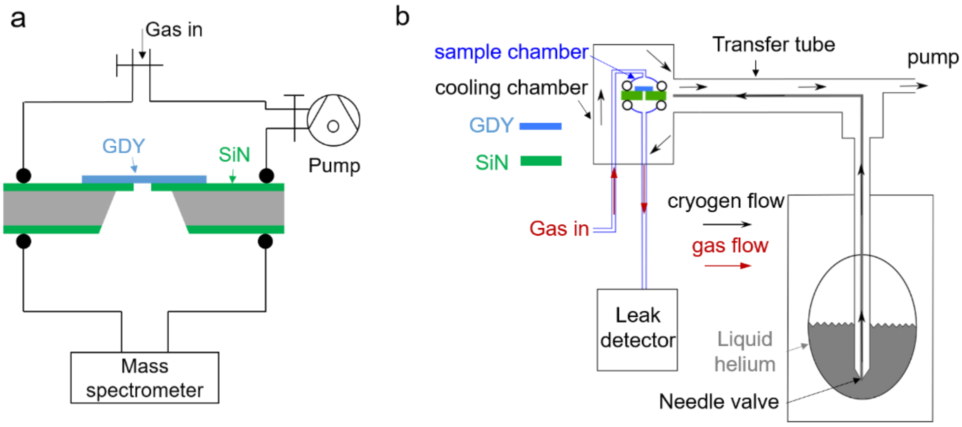

Supplementary Figure 4 | Schematics of gas permeation measurements set-up: (a) For measurements at non-cryogenic conditions. Black circles represent rubber O-rings for sealing. **(b)** For cryogenic temperature measurements. Indium seals (black empty circles next to the SiN wafer) were used instead of rubber O-rings which might fail at low temperatures.

Gas permeation through bare-hole devices

The permeance of bare-hole devices shown in Fig. 2b were measured at low feed pressure, where the mean free path of gas molecules is sufficiently large such that Knudsen flow occurs even for micro-meter sized aperture (Supplementary Figure 5a). That ensured the accuracy of our porosity estimation. In the same Knudsen regime for graphdiyne and 2 μm hole, gas flow conductance C_a through thin aperture depends linearly on the rate of impingement of molecules over pore area A ($C_a = A\sqrt{\frac{RT}{2\pi M}}$) [6]. The permeance through bare hole is ~1000 times higher than that of the graphdiyne membrane, can be therefore translated using the standard definition as 0.1% porosity.

Helium permeability through a thin aperture of 50 nm diameter in size were also measured. Here the sample is titania (monolayer 2D- Ti_xO_2) covering 2 μm SiN hole, as shown in the inset SEM image in Supplementary Figure 5b. The deviation of permeability from the inverse square root of temperature dependence at lower temperature supports the transition from Knudsen regime to

the viscous flow regime.

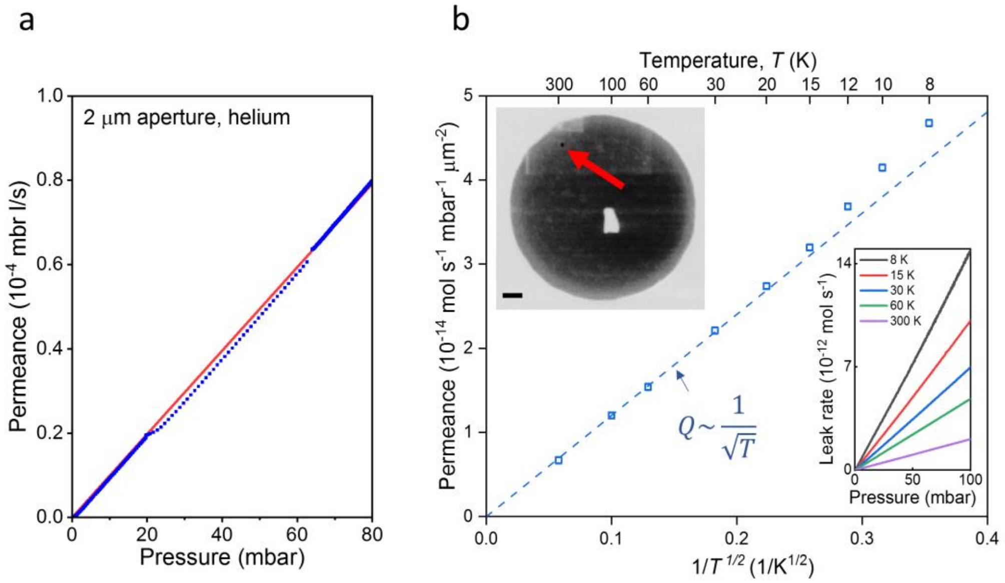

Supplementary Figure 5| Helium permeation through bare-hole devices. (a) He permeance through 2 μm aperture at low pressure. Experimental data (blue dotted line) agrees well with Knudsen prediction (red line). **(b)** Temperature dependence of He permeance through 50 nm in diameter aperture. Blue empty symbol: experimental data. Top inset shows the electron microscope image of the aperture, which appears as the dark circle indicated by the red arrow. Bottom inset shows the leak rate as a function of feed pressure at various temperatures.

DFT simulations

For the density functional theory-based first-principles calculations, the projector augmented wave[7] method was used in order to portray the pseudopotentials of C atom and the noble gas elements as implemented in Vienna Ab-initio Simulation Package (VASP)[8]. The exchange-correlation potential was taken into account by considering the local density approximation (LDA) within the Perdew-Burke-Ernzerhof (PBE) form[9]. For the geometry optimizations a kinetic energy cutoff of 500 eV was used for the plane-wave basis. The convergence criterion of the total force on each atom was reduced to 10^{-5} eV/Å and the convergence criterion of the energy was set to 10^{-6} eV.

The primitive unit cell of graphdiyne structure is composed of 18 C atoms and it was optimized using a *k*-point mesh of 18 × 18 × 1. In order to simulate the propagation of noble gas atoms through the hole of the graphdiyne structure, a super cell containing 72 C atoms was used. We checked that this was sufficiently large to avoid inter simulation cell interaction. The initial distance of a noble gas atom to the basal plane of graphdiyne was taken to be 8 Å and the vertical position of the noble gas atom was changed by 0.5 Å steps and then the total energy was calculated at each step. Note that while the rigid structure refers to the case when all the carbon atoms are frozen, the relaxed case refers to fully optimization of the whole structure as gas atoms diffuse through the lattice rings in graphdiyne.

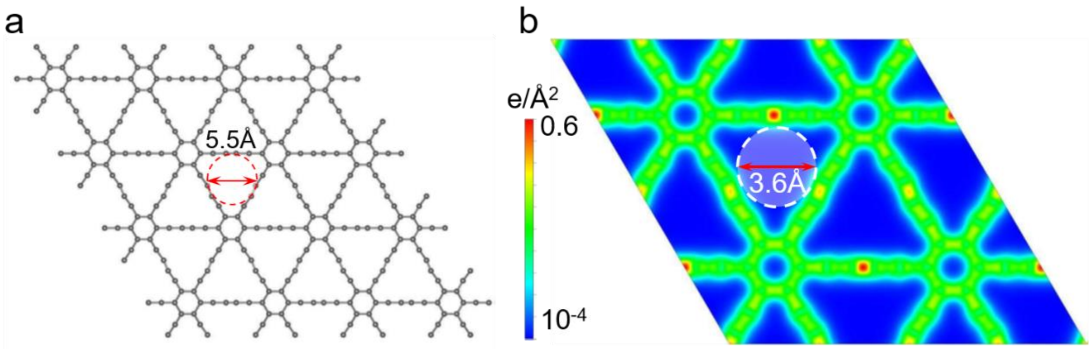

Supplementary Figure 6 | Lattice rings in graphdiyne. (a) Schematic of a monolayer graphdiyne using ball-and-stick model. The grey balls represent carbon atoms. **(b)** Graphdiyne electron density predicted by density functional theory. The electron density (in electrons per Å^2) is integrated along the direction perpendicular to graphdiyne.

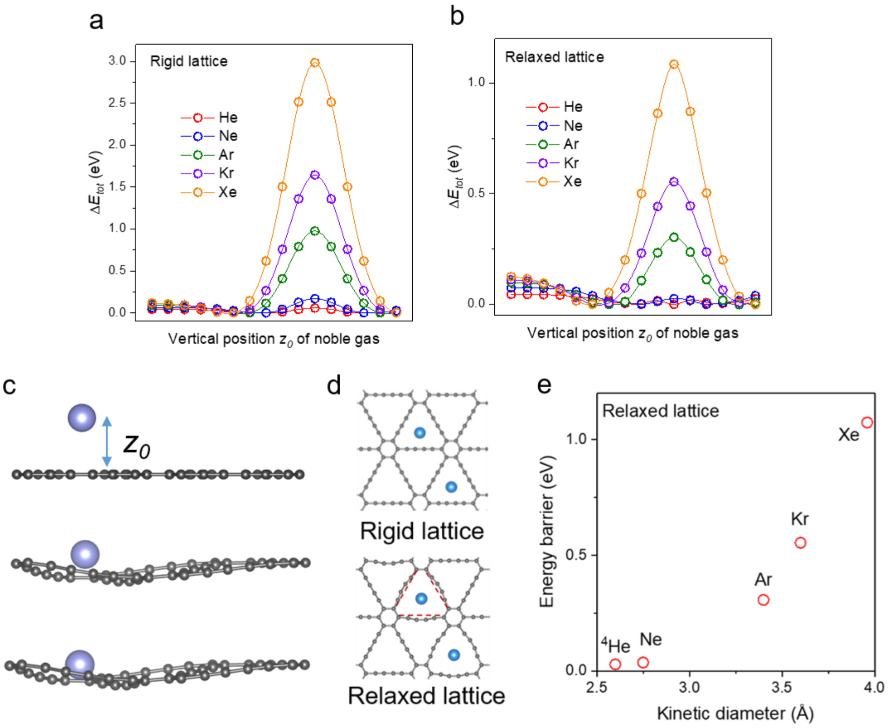

Supplementary Figure 7 | Activated gas transport through graphdiyne lattices. (a) and **(b)** Energy profiles of inert gas atoms through rigid and relaxed graphdiyne lattice rings, respectively, as a function of the vertical position z_0 between gas and graphdiyne plane. **(c)** Side view of relaxed graphdiyne lattice deformation for Xe atom (purple ball) translocation at different vertical position z_0. **(d)** Schematic of rigid and relaxed lattice when Xe atoms locate in the lattice plane, i.e. z_0 = 0. **(e)** Transport barriers of various noble gases through relaxed graphdiyne lattice.

Interaction between noble gas flows

To further demonstrate the importance of adsorption to the interacting flows between inert gases, we measured ^{4}He permeance in the ^{4}He/Ne mixture at elevated temperatures. As shown in Supplementary Figure 8, the flow rate of pure ^{4}He decreases with T as Γ_{He} ~1/√T, in consistent with the expected Knudsen flow. In contrast, though lower than Γ_{He}, helium flow rate Γ_{He+Ne} in ^{4}He + Ne

mixtures increases with T, due to the gradually weaker adsorption of Ne. As a result, at higher T, the suppression of helium flow by the presence of Ne becomes less pronounced.

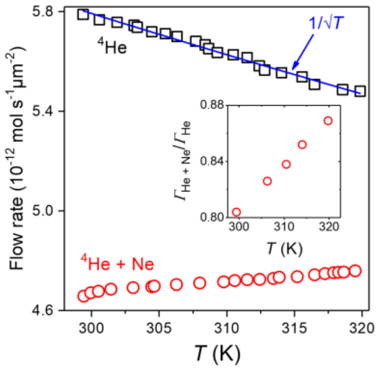

Supplementary Figure 8 | Helium permeation as a function of temperature in pure ^4He and ^4He + Ne mixtures. The partial pressure of ^4He is kept at constant of 0.2 x 10^5 Pa in both cases; Ne partial pressure is 0.8 x 10^5 Pa in binary mixtures. Inset shows the ratio of suppressed ^4He flow rate in the mixture compare to that of pure ^4He.

Comparison of permeance-selectivity tradeoff with other gas selective membranes

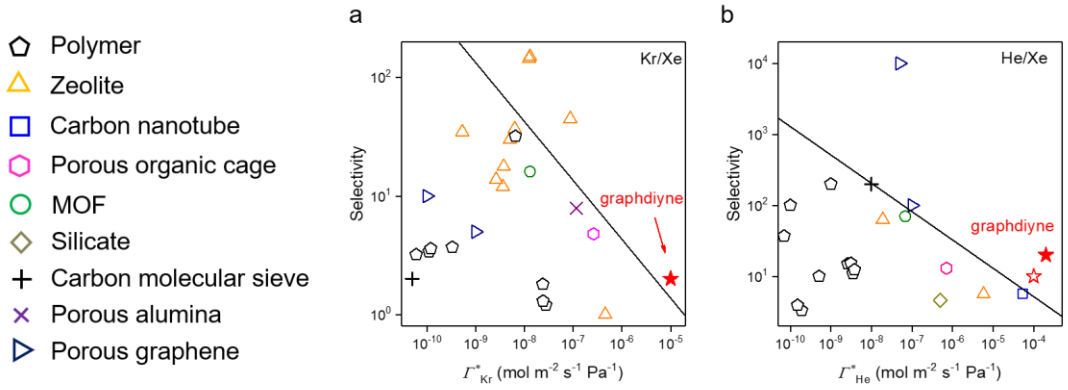

Supplementary Figure 9 | Selectivity – permeance upper bound relations for various membranes. **(a)** Kr-Xe separation. **(b)** He-Xe separation. Solid black lines indicate the state-of-art boundary, determined by the authors using gas permeation data from literatures. Filled red star represents the separation performance from the data of single component gas transport through graphdiyne-based membranes; for binary mixtures, both the permeance and selectivity are corrected by about 50%, as shown by the empty red star in (b). The literature data include membranes made from polymers[10-13], zeolites[14-20], carbon nanotubes[21], porous organic cages[22], metal organic frameworks (MOF)[23,24], silicate[25], carbon molecular sieves[26] and porous alumina[17]. All data points are experimental results with no corrections/assumptions made to membrane thickness and pore density. The figure also includes results from previously reported porous graphene. For those of perforated graphene with single-nanopores[27], we assume their pore density of 10^{12} cm^{-2} (which is the highest density currently achievable by top-down methods) without loss of single-pore functionality.

References

1. G. Li, Y. Li, H. Liu, Y. Guo, Y. Li, D. Zhu, Architecture of graphdiyne nanoscale films. *Chem. Commun.* **46**, 3256–3258 (2010).
2. X. Gao, H. Liu, D. Wang, J. Zhang, Graphdiyne: synthesis, properties, and applications. *Chem. Soc. Rev.* **48**, 908-936 (2019).
3. J. W. Suk, A. Kitt, C. W. Magnuson, Y. Hao, S. Ahmed, J. An, A. K. Swan, B. B. Goldberg, R. S. Ruoff, Transfer of CVD-grown monolayer graphene onto arbitrary substrates. *ACS Nano* **5**, 6916-6924 (2011).
4. S. Hu, M. Lozada-Hidalgo, F. C. Wang, A. Mishchenko, F. Schedin, R. R. Nair, E. W. Hill, D. W. Boukhvalov, M. I. Katsnelson, R. A. W. Dryfe, I. V. Grigorieva, H. A. Wu, A. K. Geim, Proton transport through one-atom-thick crystals, *Nature* **516**, 227-230 (2014).
5. R. R. Nair, P. Blake, J. R. Blake, R. Zan, S. Anissimova, U. Bangert, A. P. Golovanov, S. V. Morozov, A. K. Geim, K. S. Novoselov, T. Latychevskaia, *Appl. Phys. Lett.* **97**, 153102 (2010).
6. R.G. Livesey, *Flow of Gases Through Tubes and Orifices*, in *Foundations of vacuum science and technology*, J.M. Lafferty, Editor. 1998, Wiley: New York.
7. P. E. Blöchl, Projector augmented-wave method. *Phys. Rev. B* **50**, 17953-17979 (1994).
8. G. Kresse, J. Furthmüller, Efficient iterative schemes for ab initio total-energy calculations using a plane-wave basis set. *Phys. Rev. B* **54**, 11169-11186 (1996).
9. J. P. Perdew, K. Burke, M. Ernzerhof, Generalized gradient approximation made simple. *Phys. Rev. Lett.* **77**, 3865-3868 (1996).
10. S. Yu, S. Li, H. Wang, C. Zhu, J. Hou, S. Cui, X. Shen, Y. Liu, Crosslinked microporous polyarylate membranes with high Kr/Xe separation performance and high stability under irradiation. *J. Membr. Sci.* **611**, 118280 (2020).
11. Y. Nakai, H. Yoshimizu, Y. Tsujita, Enhanced gas permeability of cellulose acetate membranes under microwave irradiation. *J. Membr. Sci.* **256**, 72-77 (2005).
12. I. N. Beckman, D. A. Syrtsova, M. G. Shalygin, P. Kandasamy, V. V. Teplyakov, Transmembrane gas transfer: mathematics of diffusion and experimental practice. *J. Membr. Sci.* **601,**117737 (2020).
13. J. D. Leroux, V. V. Teplyakov, D. R. Paul, Gas-transport properties of surface fluorinated poly(vinyltrimethylsilane) films and composite membranes. *J. Membr. Sci.* **90**, 55-68 (1994).
14. X. H. Feng, Z. W. Zong, S. K. Elsaidi, J. B. Jasinski, R. Krishna, P. K. Thallapally, M. A. Carreon, Kr/Xe separation over a chabazite zeolite membrane. *J. Am. Chem. Soc.* **138**, 9791-9794 (2016).
15. Y. H. Kwon, C. Kiang, E. Benjamin, P. Crawford, S. Nair, R. Bhave, Krypton-xenon separation properties of SAPO-34 zeolite materials and membranes. *AICHE J* **63**, 761-769 (2017).
16. Y. H. Kwon, B. Min, S. Yang, D. Y. Koh, R. R.Bhave, S. Nair, Ion-exchanged SAPO-34 membranes for krypton-xenon separation: control of permeation properties and fabrication of hollow fiber membranes. *ACS Appl. Mater. Interfaces* **10**, 6361-6368 (2018).
17. T. Wu, J. Lucero, Z. Zong, S. K. Elsaidi, P. K. Thallapally, M. A. Carreon, Microporous crystalline membranes for Kr/Xe separation: comparison between AlPO-18, SAPO-34, and ZIF-8. *ACS Appl. Nano Mater.* **1**, 463-470 (2018).
18. X. R. Wang, T. Zhou, P. Zhang, W. F. Yan, Y. G. Li, L. Peng, D. Veerman, M. Y. Shi, X. H. Gu, F. Kapteijn, High-Silica CHA zeolite membrane with ultra-high selectivity and irradiation stability for Krypton/Xenon separation. *Angew. Chem. Int. Ed.* **60**, 9032-9037 (2021).
19. X. Wang, Y. Zhang, X. Wang, E. Andres-Garcia, P. Du, L. Giordano, L. Z. Wang, Hong, X. Gu, S.

Murad, F. Kapteijn, Xenon recovery by DD3R zeolite membranes: application in anaesthetics. *Angew. Chem. Int. Ed.* **58**, 15518-15525 (2019).

20. X. Wang, P. Karakilic, X. Liu, M. Shan, A. Nijmeijer, L. Winnubst, J. Gascon, F. Kapteijn, One-Pot synthesis of high-flux b-oriented MFI zeolite membranes for Xe recovery. *ACS Appl. Mater. Interfaces* **10**, 33574-33580 (2018).
21. J. K. Holt, H. G. Park, Y. M. Wang, M. Stadermann, A. B. Artyukhin, C. P. Grigoropoulos, A. Noy, O. Bakajin, Fast mass transport through sub-2-nanometer carbon nanotubes. *Science* **312**, 1034-1037 (2006).
22. J. M. Lucero, M. A. Carreon, Separation of light gases from xenon over porous organic cage membranes. *ACS Appl. Mater. Interfaces* **12**, 32182-32188 (2020).
23. T. Wu, X. Feng, S. K. Eisaidi, P. K. Thallapally , M. A. Carreon, Zeolitic imidazolate framework-8 (ZIF-8) membranes for Kr/Xe separation. *Ind. Eng. Chem. Res.* **56**, 1682-1686 (2017).
24. L. Dumee, L. He, M. Hill, B. Zhu, M. Duke, J. Schuetz, F. She, H. S. Wang, Gray, P. Hodgson, L. Kong, Seeded growth of ZIF-8 on the surface of carbon nanotubes towards self-supporting gas separation membranes.*J. Mater. Chem. A* **1**, 9208-9214 (2013).
25. W. J. W. Bakker, L. J. P. Vandenbroeke, F. Kapteijn, J. A. Moulijn, Temperature dependence of one-component permeation through a silicalite-1 membrane. *AICHE J* **43**, 2203-2214 (1997).
26. P. G. Crawford, Zeolite membranes for the separation of krypton and xenon from spent nuclear fuel reprocessing off-gas, Master Thesis, Georgia Institute of Technology (2013).
27. P. Z. Sun, M. Yagmurcukardes, R. Zhang, W. J. Kuang, M. Lozada-Hidalgo, B. L. Liu, H. M. Cheng, F. C. Wang, F. M. Peeters, I. V. Grigorieva, A. K. Geim, Exponentially selective molecular sieving through angstrom pores. *Nat. Commun.* **12**, 7170 (2021).
